# Imagem guiada para qualidade de vida de mulheres com cancer cervical: estudo quase experimental*

**DOI:** 10.15649/cuidarte.2358

**Published:** 2023-05-27

**Authors:** Edenice de Oliveira Santana, Larissa Marcondes, Luana Aparecida Alves da Silva, Namie Okino Sawada, Luciana Martins da Rosa, Luciana Puchalski Kalinke

**Affiliations:** 1 . Hospital Erasto Gaertner, Curitiba, Paraná, Brasil. Email: esanttana42@gma il.com Hospital Erasto Gaertner Curitiba Paraná Brasil esanttana42@gma il.com; 2 . Universidade Federal do Paraná, Curitiba, Paraná, Brasil. Email: marcondes.lari@gmail.com Universidade Federal do Paraná Universidade Federal do Paraná Curitiba Paraná Brazil marcondes.lari@gmail.com; 3 . Complexo Hospital de Clínicas da Universidade Federal do Paraná, Curitiba, Paraná, Brasil. Email: aas.luana@gmail.com Complexo Hospital de Clínicas da Universidade Federal do Paraná Curitiba Paraná Brasil aas.luana@gmail.com; 4 . Universidade Federal de Alfenas. Alfenas, Minas Gerais, Brasil. Email: namie.sawada@unifal-mg.edu.br Universidade Federal de Alfenas Universidade Federal de Alfenas Alfenas Minas Gerais Brazil namie.sawada@unifal-mg.edu.br; 5 . Universidade Federal de Santa Catarina. Florianópolis, Santa Catarina, Brasil. Email: luciana.m.rosa@ufsc.br Universidade Federal de Santa Catarina Universidade Federal de Santa Catarina Florianópolis Santa Catarina Brazil luciana.m.rosa@ufsc.br; 6 . Universidade Federal do Paraná, Curitiba, Paraná, Brasil. Email: kalinkeluciana@gmail.com Universidade Federal do Paraná Universidade Federal do Paraná Curitiba Paraná Brazil kalinkeluciana@gmail.com

**Keywords:** Qualidade de Vida, Neoplasias do Colo de Útero, Imaginado, Terapia de Relaxamento, Terapia de Exposiáo a Realidade Virtual, Terapias Complementares, Quality of Life, Uterine Cervical Neoplasms, Imagination, Relaxation Therapy, Virtual Reality Exposure Therapy, Complementary Therapies, Calidad de Vida, Neoplasias del Cuello Uterino, Imaginación, Terapia por Relajación, Terapia de Exposición Mediante Realidad Virtual, Terapias Complementarias

## Abstract

**Introdujo:**

As terapias mente-corpo, como o relaxamento com imagem guiada, que estáo entre as principais práticas integrativas utilizadas por pacientes oncológicos, sao essenciais para manter equilibrio saudável entre a excitado simpática e parassimpática no cérebro.

**Objetivo:**

Avaliar o efeito do relaxamento por imagem guiada, com o uso da realidade virtual, na qualidade de vida relacionada a saúde de mulheres com cancer de colo uterino em tratamento concomitante com quimiorradioterapia.

**Materiais e Método:**

Estudo quase experimental, realizado em um hospital de referencia em oncologia no Sul do Brasil, de outubro de 2019 a janeiro de 2021. Participaram 52 mulheres, divididas em dois grupos, experimental (intervengo relaxamento por imagem guiada com realidade virtual), e controle (rotina padráo). Utilizou-se para avaliaáo dos escores da qualidade de vida relacionada a saúde o instrumento Functional Assessmentof Cancer Therapy Cervix Cancer, aplicado antes, 14 dias após e, ao término do tratamento, em aproximadamente 35 dias. As análises foram realizadas pelo modelo linear generalizado misto, com a matriz de covariancia autorregressiva de ordem 1, e a significancia foi confirmada pelo teste de Sidak.

**Resultados:**

Houve diferena estatisticamente significativa para o grupo experimental nos domínios físico (p=0,02), funcional (p=0,00), sintomas específicos do cancer de colo de útero e efeitos do tratamento (p=0,03) e qualidade de vida global (p=0,02).

**Conclusáo:**

O relaxamento por Imagem Guiada contribuiu na melhora na Qualidade de Vida Relacionada a Saúde das pacientes com cancer de colo do útero, durante o tratamento com quimiorradiaáo.

## Abstract (en)

**Introduction:**

Mind-body therapies, such as guided imagery relaxation, which are among the main integrative practices used by cancer patients, are essential for maintaining a healthy balance between sympathetic and parasympathetic arousal in the brain.

**Objective:**

To evaluate the effect of guided image relaxation, with the use of virtual reality, on the health-related quality of life of women with cervical cancer undergoing concomitant treatment with chemoradiotherapy.

**Materials and Method:**

A quasi-experimental study, carried out in an oncology referral hospital in southern Brazil, from October 2019 to January 2021. 52 women participated, divided into two groups, experimental (relaxation intervention guided by imagery with virtual reality), and control (default routine). The Functional instrument was used to assess health-related quality of life scores. Assessment of Cancer therapy cervix cancer, applied before, 14 days after and at the end of treatment, in approximately 35 days. The analyzes were performed using the mixed generalized linear model, with an autoregressive covariance matrix of order 1, and the significance was confirmed by the Sidak test.

**Results:**

There was a statistically significant difference for the experimental group in the physical (p =0.02), functional (p =0.00), specific symptoms of cervical cancer and treatment effects (p =0.03) and overall quality of life (p =0.02).

**Conclusion:**

Guided Image Relaxation contributed to an improvement in the Health- Related Quality of Life of patients with cervical cancer during treatment with chemoradiation.

## Resumen

**Introducción:**

Las terapias de mente y cuerpo, como la relajación de imágenes guiadas, que se encuentran entre las principales prácticas integradoras utilizadas por los pacientes con cáncer, son esenciales para mantener un equilibrio saludable entre la activación simpática y parasimpática en el cerebro.

**Objetivo:**

Evaluar el efecto de la relajación de imagen guiada, con el uso de realidad virtual, sobre la calidad de vida relacionada con la salud de mujeres con cáncer de cuello uterino en tratamiento concomitante con quimiorradioterapia.

**Materiales y Método:**

Estudio cuasi-experimental, realizado en un hospital de referencia de oncología en el sur de Brasil, de octubre de 2019 a enero de 2021. Participaron 52 mujeres, divididas en dos grupos, experimental (intervención de relajación guiada por imaginería con realidad virtual), y control (rutina por defecto). El instrumento funcional se utilizó para evaluar las puntuaciones de calidad de vida relacionada con la salud. Evaluación de Cáncer terapia cuello uterino cáncer, aplicado antes, 14 días después y al final del tratamiento, en aproximadamente 35 días. Los análisis se realizaron utilizando el modelo lineal generalizado mixto, con una matriz de covarianza autorregresiva de orden 1, y la significación fue confirmada por la prueba de Sidak.

**Resultados:**

hubo una diferencia estadísticamente significativa para el grupo experimental en la calidad de vida física (p = 0,02), funcional (p = 0,00), síntomas específicos del cáncer de cuello uterino y efectos del tratamiento (p = 0,03) y en general (p = 0,02).).

**Conclusión:**

La Relajación por Imagen Guiada contribuyó a mejorar la Calidad de Vida Relacionada con la Salud de las pacientes con cáncer de cuello uterino durante el tratamiento con quimiorradioterapia.

## Introdujo

O cáncer de colo de útero (CCU) é considerado o quarto tipo mais comum entre as mulheres, sendo diagnosticados aproximadamente 570 mil casos novos por ano no mundo^1^. No Brasil, o Instituto Nacional do Cáncer José de Alencar Gomes da Silva (INCA) estimou, para cada ano do trienio 2020/2022, 16.590 casos novos, com um risco estimado de 15,43 casos a cada 100 mil mulheres, ocupando a terceira posigáo do ranking nacional^2^.O tratamento do CCU é realizado principalmente por cirurgia ou radioterapia, utilizando a quimioterapia como um adjuvante.

O planejamento do tratamento do CCU deve ser realizado de forma multidisciplinar, baseado no conhecimento preciso e amplo a respeito dos fatores prognósticos e preditivos para desfecho oncológico, morbidade e qualidade de vida (QV)^3^. A World Health Organization definiu QV como: "percepgáo do individuo e de sua posigáo na vida, no contexto de sua cultura e dos sistemas de valores em que vive em relagáo as suas expectativas, seus padróes e suas preocupares"^4^. Contudo, as diferentes modalidades de tratamento aplicadas para este cáncer trazem impactos negativos na Qualidade de Vida Relacionada a Saúde (QVRS), sendo este conceito adotado por estudiosos como a visáo que o individuo tem sobre sua saúde e o impacto dela na própria vida, e o quanto a doenga ou estado crónico sáo percebidos pelo individuo, ou seja, o estado de saúde percebido^5^^-^^6^.

O uso de instrumentos validados que mensurem a QVRS se tornou frequente e necessário, para se conseguir atingir a percepgáo do individuo. Entre os instrumentos desenvolvidos por agencias internacionais utilizados para avaliar a QVRS de pacientes com cáncer, pode-se destacar os questionários elaborados pela Facit.org (FACIT - Functional Assessment of Chronic Illness Therapy), organizado que gerencia a distribuido e informagóes relacionadas a mais de 100 questionários que medem a QVRS de pessoas com doengas crónicas, sáo vastamente utilizados^7^.

O entendimento da QVRS por parte do paciente e profissionais é fundamental para uma escolha conjunta das medidas terapeuticas que seráo utilizadas, observando caso a caso e visando minimizar os efeitos do cáncer e do tratamento. Pacientes que se submetem ao tratamento combinado de quimioterapia e radioterapia (CCRT) podem apresentar reagóes como: alteragóes hematológicas, náuseas, vómitos, diarreia, problemas pós-radiagáo precoce em trato gastrointestinal e bexiga^8^. Assim como estresse significativo, angústia existencial, depressáo, ansiedade, insónia, tanto durante o tratamento, quanto na sobrevivencia^9^. Outra situagáo, frequentemente evidenciada durante o tratamento, é o sentimento da perda da feminilidade e problemas sexuais e/ou emocionais^10^. Na tentativa de aliviar ou reduzir estes e outros sintomas persistentes da doenga e efeitos colaterais decorrentes do tratamento, os pacientes muitas vezes se voltam para as práticas integrativas e complementares em saúde (PICS).

As PICS demonstraram eficácia no tratamento de efeitos colaterais comuns relacionados ao cáncer, melhorando a QVRS dos pacientes. O uso de práticas como acupuntura, auriculoterapia, reike, toque terapeutico, relaxamento muscular progressivo associado a imagem guiada tem efeitos sobre os biomarcadores, como a fungáo imunológica e os hormónios do estresse, redugáo de dor, náuseas, vómitos, fadiga, falta de apetite, além de agir nas fungóes emocionais e sociais para pacientes com cáncer ginecológico^11^. As terapias mente-corpo estáo entre as principais PICS utilizadas por pacientes oncológicos, sáo essenciais para manter equilibrio saudável entre a excitagáo simpática e parassimpática no cérebro, portanto, ajudam a gerir melhor a resposta ao estresse^12^. A imagem guiada é uma intervengáo mente-corpo, que usa a própria imaginagáo e o processamento mental do paciente para a formagáo de uma representado mental de um objeto, lugar, evento ou situado percebida, por meio dos sentidos^13^.

Estudos^14^^-^^15^ demonstram a melhora na qualidade de vida, fundo emocional, social e diminuido de sintomas como fadiga e perda de apetite de pacientes com cáncer que utilizam o relaxamento com imagem guiada como PICS. No contexto de que as pacientes CCU tém sua QVRS alterada durante o tratamento, surge a hipótese deste estudo: a PICS relaxamento com imagem guiada por realidade virtual, aplicada durante o tratamento CCRT, proporciona melhora na QVRS das mulheres com CCU.

No contexto de confirmar ou refutar a hipótese da presente pesquisa, teve-se como objetivo: avaliar o efeito do relaxamento com imagem guiada, com o uso da realidade virtual, na QVRS de mulheres com CCU em tratamento concomitante com quimiorradioterapia.

## Materiais e Método

Trata-se de um estudo quase experimental^16^, realizado em hospital do Sul do Brasil, referéncia para diagnóstico e tratamento do cáncer, no período de outubro de 2019 a janeiro de 2021. A amostra foi composta por mulheres diagnosticadas com CCU, em tratamento combinado com quimioterapia e radioterapia, nas modalidades tele e braquiterapia, com alta taxa de dose (HDR), submetidas ao tratamento CCRT, maiores de 18 anos. Foram excluidas pacientes em reirradiado pélvica, hospitalizadas, em tratamento para depressáo e/ou diagnóstico de epilepsia e em uso contínuo de medicamentos psicotrópicos, com déficits motores ou cognitivos, déficits ou alteragóes visuais ou auditivas (registrados no prontuário). Foram considerados como critérios de descontinuidade: pacientes que necessitaram receber drogas antidepressivas e ansiolíticas, durante o período de estudo, desistiram do tratamento ou tiverem o tratamento interrompido, ou que náo realizassem mais de uma sessáo de relaxamento na semana.

O cálculo amostral baseou-se numa estratégia simples de dimensionamento, foi considerada a média de atendimentos de mulheres com CCU que realizaram quimiorradioterapia nos anos de 2016, 2017 e 2018, com margem de erro 5% e nível de confianga de 95%. A amostra inicial proposta foi de 71 pacientes. Entretanto, devido a pandemia de COVID-19 (Corona Virus Disease), houve redugáo do número de casos para tratamento no cenário do estudo. A coleta de dados, inicialmente prevista até outubro de 2020, foi ampliada até janeiro de 2021, quando se interrompeu a selegáo e inclusáo de participantes. Assim, o número de inclusóes totalizou 52 mulheres.

A randomizagáo foi realizada com uso de envelopes contendo as siglas GE (Grupo experimental) e GC (Grupo controle), lacrados e numerados de 1 a 71, sendo 36 com siglas para GE e 35 para GC. As pacientes foram convidadas a escolher um dos envelopes, identificando seu grupo.

As participantes do GC receberam tratamento padráo (quimioterapia, 28 sessóes de teleterapia e quatro insergóes de braquiterapia), e foram avaliadas quanto a QVRS trés vezes durante o tratamento, no primeiro dia (basal), 14 dias após inicio, e ao término do tratamento com aproximadamente 35 dias.

A intervengáo aconteceu em uma sala reservada antes das sessóes de teleterapia ou braquiterapia, com duragáo de aproximadamente 10 minutos. O relaxamento foi composto de video de imagens de natureza, fundo musical e voz suave que conduzia a paciente ao relaxamento, proporcionando uma experiéncia sensorial para resposta clinica. Utilizou-se a combinagáo de relaxamento muscular progressivo (RMP), para indugáo de estado de relaxamento, seguida da imagem guiada com projegáo de imagens em 360°. As pacientes receberam previamente demonstrado quanto ao uso dos óculos, e do manuseio do controle para a experiencia em realidade virtual. A pesquisadora acompanhou as pacientes durante todas as sessóes de relaxamento.

As participantes do GE foram submetidas, em média, a 12 sessóes de relaxamento com imagem guiada^17^^-^^18^ com uso da realidade virtual, na frequencia de tres vezes na semana durante a sessáo CCRT, considerado o tratamento padráo. A avaliagáo da QVRS ocorreu na mesma periodicidade do grupo controle.

Os grupos GE e GC responderam questionários elaborados pelas pesquisadoras com variáveis sociodemográficas e clínicas, sendo elas idade, escolaridade, estado civil, ocupado, tipo histológico, estadiamento (conforme o sistema proposto pela International Federation of Gynecology and Obstetrics (FIGO), tempestividade, renda familiar, modalidade de radioterapia realizada e protocolo de quimioterapia realizada. Para avaliagáo da QVRS foi utilizado o questionário Functional Assessment of Cancer Therapy Cervix Cancer (FACT- Cx)^19^, disponível em portugués, após autorizado grupo norte americano Facit.org (FACIT -Functional Assessment of Chronic Illness Therapy), detentora dos direitos autorais do original e da versáo traduzida para o Brasil. É composto por um módulo geral com 27 itens que avaliam a QV geral Functional Assessment of Cancer Therapy - General (FACT-G) e um módulo denominado "preocupagóes adicionais", contendo 15 itens que avaliam sintomas relacionados a regiáo ginecológica.

Os dados coletados foram armazenados eletronicamente, em planilhas Microsoft Excel Office 365®. As informagóes validadas foram exportadas para o pacote estatístico Stata/MP versáo 14.0. para processamento de dados. O banco de dados foi armazenado no Harvard Dataverse^20^. Os resultados da caracterizagáo sociodemográfica e clínica foram submetidos a análises descritivas, com distribuigáo e frequéncia simples e absoluta. A análise dos dados da QVRS se deu conforme orientagóes do FACIT Measurement System (FACT-Cx Scoring Guidelines (Version 4), guia disponibilizado pela organizagáo em que quanto maior o escore encontrado, melhor a QVRS, sendo considerados na interpretagáo os escores reversos dos itens negativos. A pontuagáo do questionário é obtida por escala do tipo Likert de 0 (nem um pouco) a 4 (muitíssimo). O escore varia de 0 a 168, quanto mais elevada a pontuagáo, melhor a QVRS^21^.

As diferengas entre grupos e etapas na avaliagáo da QVRS foram realizadas por meio do modelo linear generalizado misto (GLMM), que permite conduzir análises com dados de participantes com seguimento interrompido, onde as medidas náo precisam ser igualmente espagadas e balanceadas^22^. O ajuste do modelo definiu-se pelo Akaike's Information Criterion (AIC), e foi utilizada a matriz de covariancia autorregressiva de ordem 1 (AR1). As diferengas significativas foram confirmadas pelo teste de Sidak.

O estudo atendeu a Resolugáo 466/12 do Conselho Nacional de Saúde, do Ministério da Saúde do Brasil, que regulamenta diretrizes éticas da pesquisa envolvendo seres humanos, sendo aplicado o Termo de Consentimento Livre e Esclarecido. Foi aprovado pelo Comité de Ética em Pesquisa do cenário do estudo com o Parecer n° 3.508.505.

## Resultados

**Figura 1:**
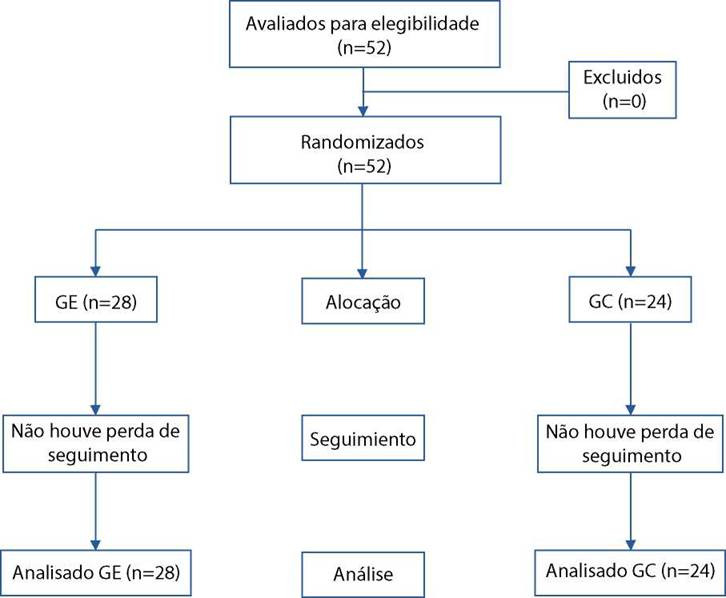
Fluxo dos participantes envolvidos no estudo. Curitiba/PR, Brasil, 2021

Participaram deste estudo 52 mulheres com cancer de colo de útero que estavam em tratamento CCRT, sendo alocadas 28 no GE e 24 no GC, conforme a Figura 1.

A idade média das participantes foi de 41,96 anos no GC e 48,82 anos para GE, havendo predominancia na faixa etária de 24 a 39 anos, em ambos os grupos (GC=58,33%, GE=35,71%). Em relagáo ao estado civil, 41,67% (n=10) de pacientes do GC eram solteiras e 42,85% (n=12) do GE eram casadas ou com uniáo estável. A ocupagáo ativa teve predominancia para ambos os grupos, com 54,17% (n=13) e 42,85% (n=12), para os grupos GC e GE, respectivamente (Tabela 1).

O tipo histológico frequente nos grupos do estudo GC e GE foi o Carcinoma Espinocelular, sendo 95,83% (n=23) e 100% (n=28), respectivamente, em estadiamento mais avanzado da doenga, com maior número de casos IIIC no GC, com 20,83% (n=5), e 39,29% (n=11) IIIB no Ge. Em relagáo ao tipo de radioterapia a predominante para ambos os grupo GC e GE, foi na modalidade 3D, sendo 95,83% (n=23) e 89,28% (n=25) respectivamente. A quimioterapia utilizada foi a cisplatina 91,67% (n=22) para o GC e 92,87% (n=26) para o GE. A tempestividade náo atendeu aos critérios estabelecidos de prazo de 60 dias para início do tratamento em 58,33% (n=14) para o GC, e 64,28% (n=18) para o GE, conforme descrito na Tabela 1.

**Tabela 1:** Distribuido de mulheres com cáncer de colo de útero em tratamento de quimioradioterapia, dos grupos controle e experimental, segundo variáveis sociodemográficas e clínicas. Curitiba/PR, Brasil, 2021 (n=52)

Variável	Grupo controle (GC)	Grupo experimental (GE)
	% (n=24)	% (n=28)
Idade em anos		
24 a 39	58,33 (14)	35,71 (10)
40 a 54	25,00 (6)	21,42 (6)
55 a 69	16,67 (4)	28,57 (8)
70 a 84	0 (0)	14,28 (4)
Ocupaao		
Assalariada	54,17 (13)	42,85 (12)
Trabalha em casa	8,33 (2)	21,42 (6)
Aposentada	12,50 (3)	21,42 (6)
Desempregada	25,00 (6)	14,28 (4)
Estado Civil		
Solteira	41,67 (10)	32,14 (9)
Casada ou Uniao estável	29,17 (7)	42,85 (12)
Separada ou Divorciada	16,67 (4)	17,85 (5)
Viúva	12,50 (3)	7,14 (2)
Escolaridade		
Nao estudou	0 (0)	10,71 (3)
Fundamental	29,17 (7)	39,28 (11)
Médio	58,33 (14)	39,28 (11)
Graduado	12,50 (3)	10,71 (3)
Renda familiar*		
Até 1 salário-mínimo	0 (0)	10,71 (3)
1 a 3 salários-mínimos	95,83 (23)	75,00 (21)
3 a 10 salários-mínimos	4,17 (1)	14,28 (4)
Tipo Histológico		
Carcinoma espinocelular	95,83 (23)	100 (28)
Adenocarcinoma	4,17 (1)	0 (0)
Estadiamento		
IB1	4,17 (1)	0 (0)
IB2	8,33 (2)	3,71 (1)
IIA	4,17 (1)	10,71 (3)
IIIA	4,17 (1)	0 (0)
IIB	29,16 (7)	25,00 (7)
IIIB	20,83 (5)	39,28 (11)
IIIC	20,83 (5)	14,28 (4)
IVA	8,33 (2)	0 (0)
IVB	0 (0)	7,14 (2)
Variável	Grupo controle (GC)	Grupo experimental (GE)
	% (n=24)	% (n=28)
Tipo de Radioterapia		
2D	4,17 (1)	10,71 (3)
3D	95,83 (23)	89,28 (25)
Tipo de Quimioterapia		
Cisplatina	91,67 (22)	92,87 (26)
Carboplatina	8,33 (2)	7,14 (2)
Tempestividade em dias		
20 a 60	41,67 (10)	35,71 (10)
61 a 80	33,33 (8)	28,57 (8)
81 a 100	25,00 (6)	35,71 (10)

NOTA:* O salário mínimo vigente no país, no período do estudo, era de R$1.045, equivalente a US$189,20.

A análise da QVRS, mensurada pelo questionário FACT-G, demonstrou aumento nos escores relacionados ao bem-estar físico no GE, com escore basal de 13,70, atingindo 16,31 na terceira avaliagáo, apontando melhora. Enquanto no GC a variagáo foi inversa, com escore basal maior (15,75), com declínio na terceira avaliagáo (14,67). No bem-estar emocional o escore partiu de 14,27 e chegou 15,89 no GE, e no GC de 14,73 para 13,80 (Tabela 2).

A Qualidade de Vida Global (FACT-G/0- 1 08) apresentou média basal no GE de 54,65, chegando a 59,16 ao término do tratamento, enquanto no GC houve declínio dos escores, de média basal 51,82 para 47,38 na terceira avaliagáo, que corresponde ao final do tratamento. O escore inicial da QVRS do paciente com CCU (FACT-Cx/ 0-168) apontou o GE com valores maiores em relagáo ao GC; quando acompanhadas as pacientes entre as etapas, o GC apresentou valores mais baixos já no baseline, contudo, ao final da terceira avaliagáo, esses valores apresentaram-se menores ainda (Tabela 2).

**Tabela 2:** Média e desvio padráo das escalas do instrumento Functional Assessment Of Cancer Therapy Cervix Cancer, nos tres tempos de avaliaáo, grupo controle e grupo intervengo. Curitiba/PR, Brasil, 2021

	Etapas da Pesquisa								
Domínios	Basal			Avaliaao 2			Avaliaao 3		
	n	Média	DP	n	Média	DP	n	Média	DP
Bem-estar físico									
Grupo intervengo	27	13,70	5,07	25	16,32	5,76	24	16,31	3,32
Grupo controle	24	15,75	5,60	21	17,49	4,82	18	14,67	4,97
Bem-estar social/familiar									
Grupo intervengo	28	14,54	3,97	25	14,59	3,96	25	14,23	3,42
Grupo controle	24	12,54	4,96	21	12,96	4,86	18	10,78	4,21
Bem-estar emocional									
Grupo intervengo	28	14,27	5,05	25	15,02	4,18	24	15,89	2,34
Grupo controle	22	14,73	3,37	20	14,50	2,98	18	13,80	3,53
Bem-estar funcional									
Grupo intervengo	28	12,44	4,12	25	13,06	4,35	24	12,59	3,35
Domínios	Basal			Avaliaido 2			Avaliaido 3		
	n	Média	DP	n	Média	DP	n	Média	DP
Grupo controle Preocupares adicionais	23	9,26	4,62	20	10,05	4,42	18	8,14	2,00
Grupo intervenido	27	27,57	8,42	23	28,69	6,06	24	29,53	5,48
Grupo controle	24	25,98	9,64	21	24,93	6,11	17	22,53	5,70
TOI									
Grupo intervenido	27	53,50	13,02	23	59,02	12,98	24	58,43	10,08
Grupo Controle	23	50,02	15,45	20	52,37	11,74	17	45,80	6,82
FACT-G									
Qualidade de vida global									
Grupo intervenido	27	54,65	12,95	25	59,00	13,18	24	59,16	8,34
Grupo controle	22	51,82	13,61	20	54,75	12,30	18	47,38	7,27
FACT-G									
Cáncer de colo uterino									
Grupo intervenido	27	82,23	17,75	23	89,03	15,60	24	88,69	12,43
Grupo controle	22	77,34	21,41	21	77,07	19,56	17	70,59	9,57

*LEGENDA: DP - Desvio padráo; FACT-G - Functional Assessment of Cancer Therapy General; FACT-Cx - Functional Assessment of Cancer Therapy Cervix Cancer; TOI - Trial Outcome Index (soma dos escores de bem-estar físico, bem-estar funcional e preocupares adicionais)*

Na análise GLMM, realizada para verificar se houve diferenga significativa ao longo do tempo (basal, avaliagáo 2 e 3) e entre os grupos e etapas, os resultados apresentaram diferengas significativas entre os grupos nos domínios funcional e domínio de QVRS relacionado a sintomas do CCU (p=0,00; p=0,03, respectivamente). Estes achados demonstram que as pacientes que realizaram a intervengáo tiveram melhoras nos sintomas funcionais e sintomas específicos do CCU, e nos efeitos do seu tratamento. Desta forma, podemos confirmar que há diferenga significativa para os domínios apontados entre os grupos, conforme demonstrado na Tabela 3.

Em relagáo ao tempo, observa-se que houve diferenga estatisticamente significativa entre as etapas da coleta, para o bem-estar físico (p=0,02). Quanto ao bem-estar funcional (0-28), no que se refere as diferengas entre os tempos, observa-se significancia, quando aplicado o modelo GLMM, no entanto, náo houve confirmagáo quando aplicado o teste de Sidak.

**Tabela 3:** Detalhamento do modelo estatístico GLMM, variável dependente por domínio de qualidade de vida. Curitiba/PR, Brasil, 2021

Fonte	Testes de Efeitos Fixos Tipo III			
	DF	GL	F	P
Bem-estar físico - AIC = 810,914				
Intercepto	1	54,893	846,774	0
Grupo	1	54,893	0,239	0,62
Tempo	2	90,223	4,004	0,021
Grupo * tempo	2	90,223	1,987	0,14
Fonte	Testes de Efeitos Fixos Tipo III			
	DF	GL	F	P
Bem-estar social/familiar - AIC = 704,35				
Intercepto	1	50,367	575,772	0,00
Grupo	1	50,367	3,080	0,08
Tempo	2	85,174	1,780	0,17
Grupo ** tempo	2	85,174	0,448	0,64
Bem-estar emocional - AIC = 721,93				
Intercepto	1	50,549	1219,591	0,00
Grupo	1	50,549	0,838	0,36
Tempo	2	85,050	0,205	0,81
Grupo *** tempo	2	85,050	1,605	0,20
Bem-estar funcional - AIC = 703,14				
Intercepto	1	51,389	498,428	0,00
Grupo	1	51,389	12,960	0,001
Tempo	2	85,134	3,397	0,031
Grupo **** tempo	2	85,134	0,539	0,58
Bem-estar Cervix cáncer Subscale - AIC = 869,15				
Intercepto	1	49,167	925,396	0,00
Grupo	1	49,167	4,689	0,031
Tempo	2	79,918	0,268	0,76
Grupo ***** tempo	2	79,918	1,832	0,16

*LEGENDA: DF- numerador; GL - grau de liberdade; F - Estatística Fde Snedecor;p = significancia estatística; AIC = Critério de Informando de Akaike (melhor ajuste do modelo)NOTA: 1p=<0,05.*

## Discussao

O diagnóstico e o tratamento do CCU ocasionam impacto negativo nos diferentes domínios da QVRS das mulheres, uma vez que afetam diversos aspectos da vida. Fazer com que os pacientes aprendam a relaxar em situagóes estressantes, diminuindo a estimulado simpática e aumentando a parassimpática, é fundamental para reduzir os efeitos prejudiciais do estresse crónico, reduzindo o sofrimento e melhorando a QVRS^12^. Neste estudo quase experimental, a utilizado do relaxamento com imagem guiada por realidade virtual, associado ao RMP, durante o tratamento CCRT, resultou em melhor QVRS das pacientes que receberam a intervendo, quando comparado ao GC. A intervendo configura uma terapia de fácil aplicado, segura e de baixo custo, e se mostra efetiva para a melhoria da QVRS no contexto do cáncer.

A média geral de idade deste estudo foi semelhante ao estudo brasileiro realizado com mulheres em idade produtiva, entre 25-39 anos, em tratamento de CCU^23^. Um estudo^24^ com 173 pacientes com CCU, cujo objetivo foi avaliar a QV nas diferentes faixas etárias, demonstrou forte associagao a interferencia da QV para pacientes do grupo entre 18 e 45 anos, por estar trabalhando, ter maior grau de escolaridade e ausencia de morbidade psiquiátrica. As diferengas nas faixas etárias das pacientes com CCU podem impactar na QV, e conhecé-las contribuí para a programado das intervengóes a serem prestadas a essas mulheres^25^.

Importante observar que, para mulheres em idade produtiva, a presenga de doenga e as toxicidades dos tratamentos podem impactar no desempenho de suas atividades diárias. Outro fator a ser considerado é a situagao conjugal^26^. A compreensao das representagóes de conjugalidade dessas mulheres é relevante, uma vez que indica a intengao de um cuidado mais completo e abrangente no ámbito da saúde da mulher^27^. Os prejuízos decorrentes devem ser considerados no desenvolvimento da prática clínica, uma vez que as sequelas sexuais afetam significativamente a vida destas pacientes, além de comprometer o relacionamento com seus parceiros^28^.

Um estudo transversal, incluindo 194 mulheres com CCU entre 25-69 anos, com parceiros sexualmente ativos, demonstrou que 33% (n=64) delas tiveram pontuagao acima de corte para sofrimento sexual. Níveis de angústia sexual estiveram mais associados as disfungóes vaginais, sendo estas alteragóes amplamente presentes durante e após os tratamentos^29^. Este perfil de pacientes pode ser beneficiado com terapias mente-corpo, pois elas proporcionam bem-estar emocional, melhoraram a ansiedade, satisfagao e QV^30^. Um estudo de revisao aponta que, além da melhoria da QVRS, a utilizagao do relaxamento com imagem guiada oferece outros benefícios. Em especial, para mulheres em tratamento do cáncer, os resultados apontam evidéncias de melhora na autoestima e bem-estar, promogao do empoderamento e redugao na angústia relacionada a imagem corporal^31^.

Para a QVRS, um estudo com delineamento transversal realizado na África do Sul, que avaliou a QV de 153 pacientes com CCU, com os questionários European Organization for Researchand Treatment of Cancer (EORTC) Quality of Life Questionnaire Core 30 (QLQ-C30) e Cancer Quality of Life Questionnaire - Cervical Cancer (QLQ-Cx-24), durante o tratamento, 6 meses e 12 meses após o término, demonstrou que, apesar de melhores índices na saúde global, o CCU e seu tratamento influenciaram negativamente na QVRS, em todos os domínios da vida dessas mulheres^32^. O declínio funcional é comumente encontrado nos diferentes tipos de cáncer, uma vez que estes pacientes sofrem impactos negativos dos tratamentos para esta doenga. As toxicidades decorrentes da terapia antineoplásica podem resultar na redugao da capacidade funcional e QV destes pacientes. Assim, destaca-se a importáncia de incluir na prática clínica a avaliagao da perda da funcionalidade durante o enfretamento do cáncer^33^.

Autores^34^ adotaram como intervengao o relaxamento com imagem guiada com a utilizagao de 15 minutos de áudio ao menos duas vezes na semana, em 152 pacientes com cáncer em quimioterapia em Sao Paulo, e avaliaram a QVRS com o instrumento European Organization Research Treatment of Cancer - QLQ-C30. Foram encontradas diferengas estatisticamente significativas para fungao física, emocional, cognitiva, social, desempenho de papel e fadiga, além de achados positivos em relagao a sintomas entre as etapas de avaliagao. Demonstrando o quanto significante pode ser o uso das PICS para este perfil de paciente.

O impacto na QVRS, durante o tratamento com radiagao, afeta temporariamente o bem-estar funcional de pacientes tratadas para CCU, sendo evidenciado maior comprometimento deste domínio no final do tratamento^35^. Sintomas como dor, alteragóes funcionais e emocionais afetam significativamente a saúde global e a QVRS de pacientes em tratamento para CCU^36^.

Melhores resultados de QVRS, encontrados neste estudo, foram demonstrados nos domínios funcional e sintomas específicos do CCU, no grupo de pacientes que receberam a técnica de Relaxamento por Imagem Guiada, corroborando com os dados de um estudo que utilizou a combinagao de relaxamento muscular progressivo e imagem guiada, em pacientes com cáncer de mama e próstata, durante o tratamento quimioterápico, o qual mostrou resultados significativos na redugáo de náuseas, vómitos, dor e fadiga, e melhores escores na QVRS^37^.

Um estudo desenvolvido com 60 pacientes iranianos, com cáncer de mama e próstata, em que se utilizaram a técnica de relaxamento muscular progressivo, imagem guiada e respirado diafragmática, mostrou melhora estatisticamente significativa na QVRS e funcionamento físico, imediatamente após, e seis semanas depois da intervengo no grupo de estudo, em comparado com o controle^38^. Dados de uma revisáo sistemática, na qual foram avaliados os efeitos da combinado de Relaxamento Muscular Progressivo e Imagem Guiada na QVRS de pacientes com cáncer, durante a quimioterapia, apresentaram resultados que comprovam a eficácia desta técnica na melhora de sinais e sintomas como náuseas e vómitos e estado mental dos pacientes submetidos a esta terapia^39^.

Um ensaio clínico randomizado, em que se utilizou a combinado das técnicas empregadas neste estudo, demonstrou resultados estatisticamente significativos (p<0,0001), em que a funcionalidade e a QV global dos pacientes do grupo intervengo mudaram significativamente, ao longo do tempo e entre grupos. Os escores globais da QV tiveram aumento de 9,5 pontos no grupo que sofreu a intervengo, o contrário ocorreu no grupo controle, em que houve um decréscimo de 10,7 pontos^36^.

A terapia de relaxamento por imagem guiada combinada ao relaxamento muscular progressivo tem- se demonstrado importante adjuvante nas diferentes modalidades de tratamento do cáncer. Sinais e sintomas associados a doenga e/ou tipo de tratamento tém sido atenuados por meio desta prática, conduzindo o paciente a uma melhora nos diferentes domínios da QRVS^38^.

Como limitagáo deste estudo, destaca-se o impacto relacionado a Pandemia da COVID-19, que influenciou na oferta de vagas de radioterapia e, com isso, na redugáo de pacientes com CCU encaminhadas para o servido do estudo.

Destarte, o presente estudo contribui para a prática assistencial em enfermagem, pois reforja e intensifica a prática baseada em evidéncias, promovendo o cuidado holístico ao paciente com cáncer, e valorizando a utilizagáo das PICS na sistematizado da assisténcia de enfermagem.

## Conclusao

Evidencia-se a efetividade do relaxamento muscular progressivo com imagem guiada, em pacientes com CCU em tratamento combinado de quimiorradiagáo. As pacientes que sofreram a intervengo obtiveram melhores resultados nos domínios físicos, emocional, qualidade de vida global e no dominio em que se avaliam as alteragóes e preocupares específicas deste tipo de cáncer. Para os domínios de fungáo social/familiar e funcional no GE, náo houve redugáo de mais de um ponto dos escores, se mantendo estáveis durante o tratamento, indicando, assim, um efeito útil dessa terapia, auxiliando para manter as fungóes desses domínios estáveis, enquanto no GC houve piora durante esse processo. Sugere-se que sejam realizados mais estudos com uma amostra maior, na mesma ou novas populagóes, em um contexto fora de pandemia, para que os resultados se confirmem e possam ser generalizados.
